# Selectivity in multiple multicomponent reactions: types and synthetic applications

**DOI:** 10.3762/bjoc.15.46

**Published:** 2019-02-21

**Authors:** Ouldouz Ghashghaei, Francesca Seghetti, Rodolfo Lavilla

**Affiliations:** 1 Laboratory of Medicinal Chemistry, Faculty of Pharmacy and Food Sciences and Institute of Biomedicine (IBUB), University of Barcelona, Av. de Joan XXIII, 27-31, 08028 Barcelona, Spain; 2 Department of Pharmacy and Biotechnology (FaBiT), University of Bologna, Via Belmeloro, 6, 40126, Bologna, Italy

**Keywords:** isocyanides, multicomponent reactions, reaction discovery, selectivity

## Abstract

Multiple multicomponent reactions reach an unparalleled level of connectivity, leading to highly complex adducts. Usually, only one type of transformation involving the same set of reactants takes place. However, in some occasions this is not the case. Selectivity issues then arise, and different scenarios are analyzed. The structural pattern of the reactants, the reaction design and the experimental conditions are the critical factors dictating selectivity in these processes.

## Introduction

**Scheme 1:**
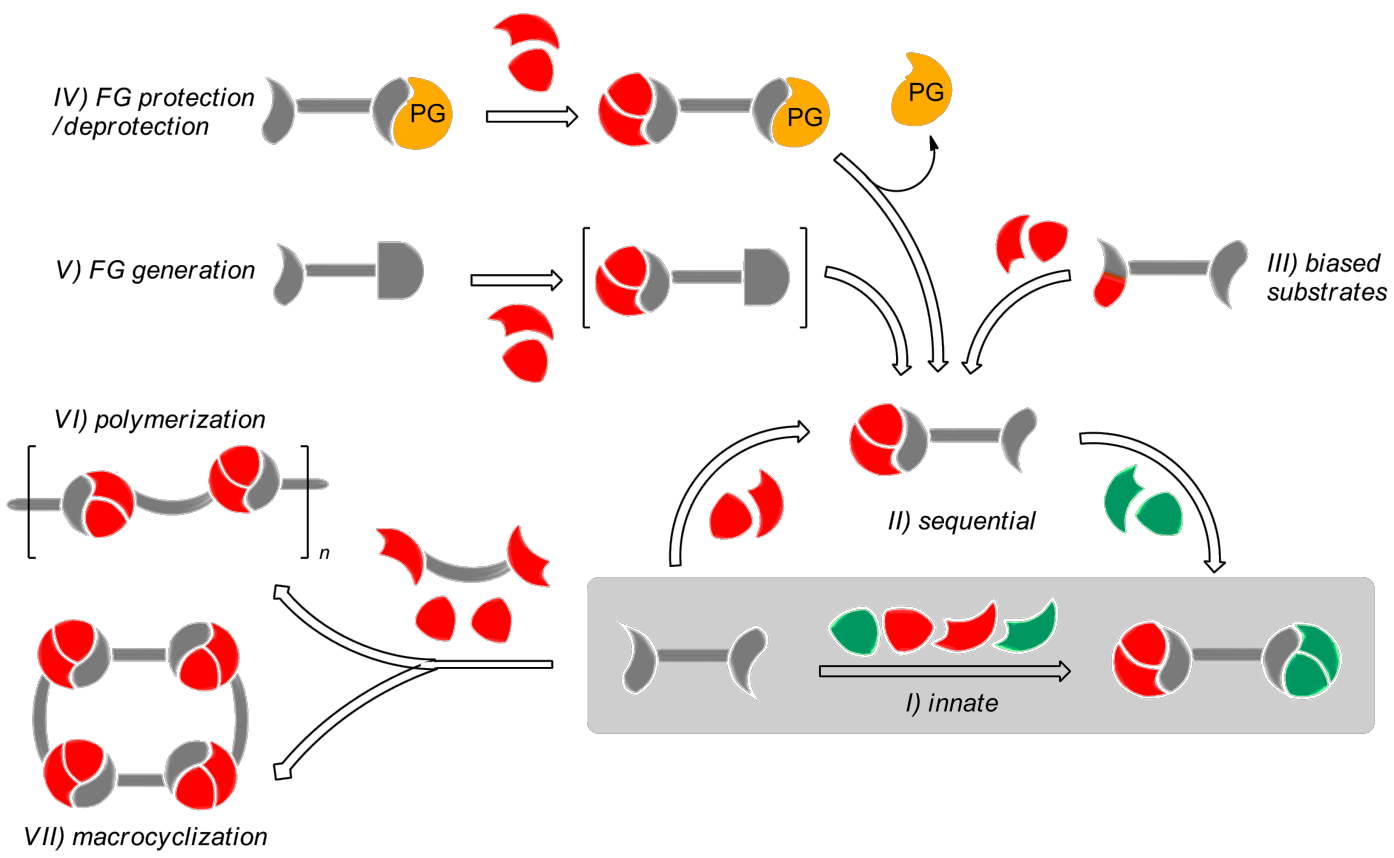
Selectivity levels found in multiple multicomponent reactions. I) Innate selectivity; II) sequential selectivity; III) use of biased substrates; IV) use of protecting groups; V) generation of new functional groups; VI) polymerization; VII) macrocyclization; FG = functional group.

Organic synthesis has become fundamental in science and technology, affecting many aspects of our lives. Therefore, there is a big need for the optimized preparation of a variety of compounds [1–3]. In this context, multicomponent reactions (MCRs) hold a privileged position, allowing the formation of many bonds and connecting three or more reactants in one step [4]. A particularly attractive and synthetically productive set of MCRs involve di/polyfunctionalized substrates that can, consequently, lead to repeated processes. The so-called multiple multicomponent reactions (MMCRs) display impressive power regarding connectivity and bond-forming efficiency and can be considered as ideal synthetic processes in many aspects [5]. These features may be maximized if the controlled incorporation of distinct reactant units, belonging to the same class, along the transformation would be feasible (Scheme 1). This review, which does not intend to be exhaustive, deals with the selectivity levels found in the literature and their impact on the synthetic outcome. In this way we may find different scenarios: i) completely unselective combinations leading to mixtures, symmetrical adducts or polymers; ii) structurally preorganized systems allowing the generation of macrocycles and iii) selective processes in which a determined combination arises in a sequential manner, either directly or indirectly, through functional group deprotection/generation. Stereoselectivity (where applicable) arising out of the individual MCRs or in the final adduct, is not contemplated. Only the connectivity issues related with the identity of the reactants are taken into account.

## Review

**Scheme 2:**
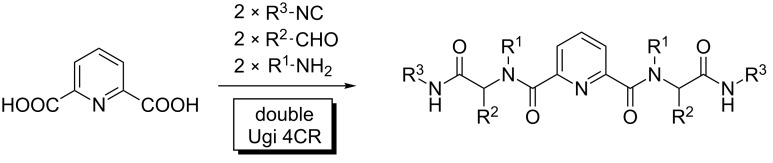
Indiscriminate double Ugi MCR upon pyridine-2,6-dicarboxylic acid.

Substrates displaying two (or more) identical functional groups (FGs) have been reacted in a variety of MCRs. An early example involves the synthesis of protease inhibitors by double Ugi 4CR using pyridine-2,6-dicarboxylic acid, isocyanides, amines and aldehydes (Scheme 2), and the combinatorial implications of this protocol were analyzed [6]. Ugi and Dömling soon realized the potential of such experiments, which helped to pave the way of combinatorial chemistry and its applications for the fast generation of pharmacological hits through library deconvolution. The reactivity of the system led to complex product mixtures, with no practical substrate selectivity.

However, if in analogous experiments several reactants of a kind, displaying different reactivities, are used, their relative nucleophilicities/electrophilicities [7] may lead, in principle, to biased mixtures. At the same time, this innate selectivity is not straightforward, as the most reactive combination would promote a fast first MCR, and likely the following MCR processes may also involve this same set [8]. Experimental findings showing complex mixtures, although far from statistical product ratios, are the usual outcome of MMCRs involving several reactants of one kind.

**Scheme 3:**
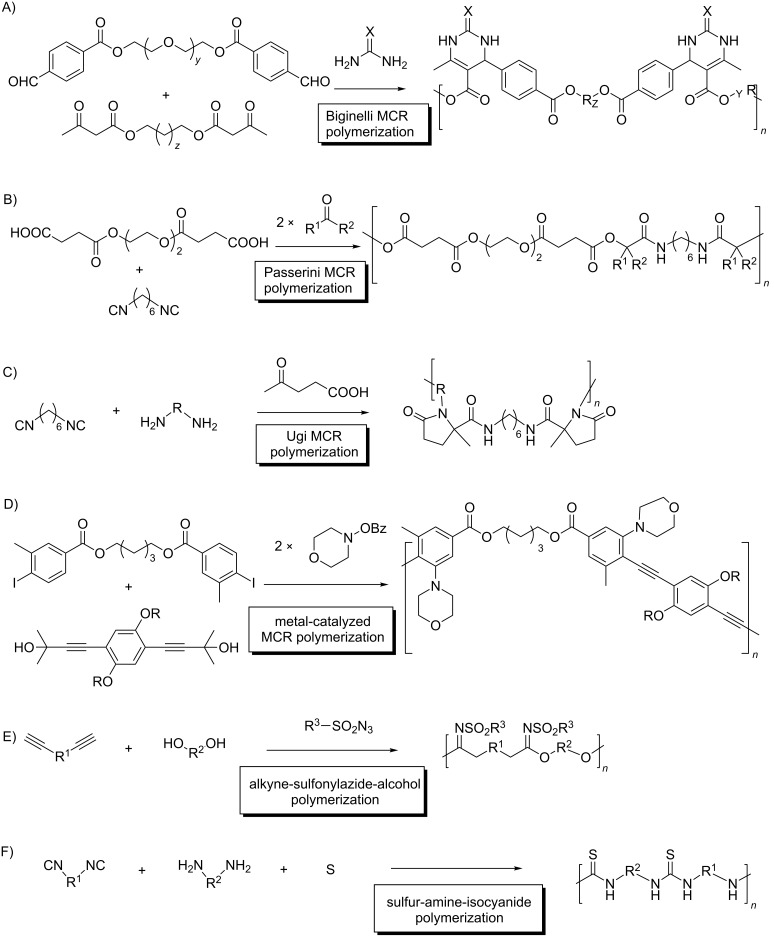
Representative examples of MCR-polymer synthesis. A) Biginelli HTS of polymers; B) Passerini;- C) Ugi-; D) metal-catalyzed MCR polymerizations; E) alkyne-sulfonylazide-alcohol interactions; F) sulfur-amine-isocyanide combination.

There are, however, cases where this indiscriminate reactivity is synthetically useful: MCR polymerizations, typically involving two doubly functionalized reactants, which yield macromolecular adducts [9–11]. In these processes, the reactivity of the equivalent FGs is nearly identical in the reactants and in the oligo/polymeric intermediates, as they are usually connected through long linear alkyl chains. Representative examples include polymerizations using Biginelli [12], Passerini [13], Ugi [14], metal-catalyzed MCRs [15], and reactive combinations involving an alkyne-sulfonyl azide nucleophile [16] and sulfur, amines and isocyanides [17] (Scheme 3).

An interesting case, where this principle does not apply, is introduced by Wessjohann [5,18], who introduced a family of MMCRs leading to complex macrocyclic structures instead of linear polymer chains. In these examples, a low concentration together with the use of specific substrates tune the reactivity pattern, promoting intramolecular interactions over intermolecular polymerizations. Thus, the use of the right conditions and the intelligent choice of the polyfunctionalized building blocks, enable an “architectural approach” towards the MMCR synthesis of macrocyclic adducts [19].

**Scheme 4:**
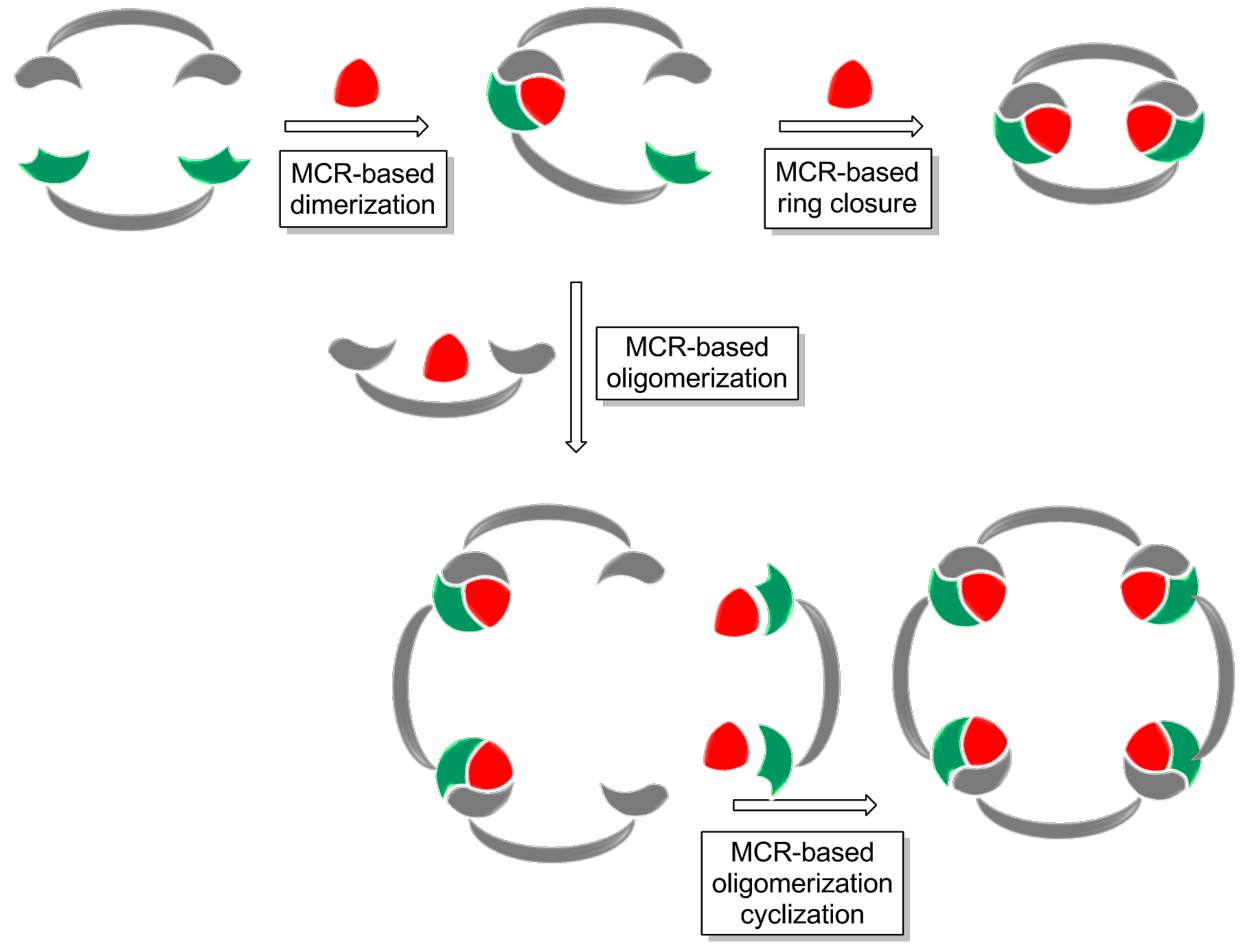
Concept of multicomponent macrocyclization.

**Scheme 5:**
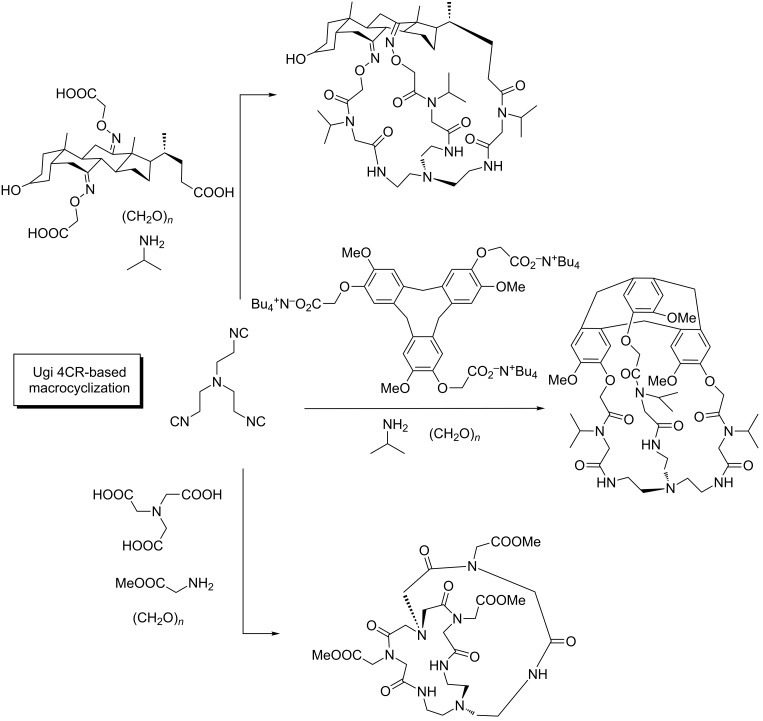
Supramolecular structures out of MMCR macrocyclizations.

**Scheme 6:**
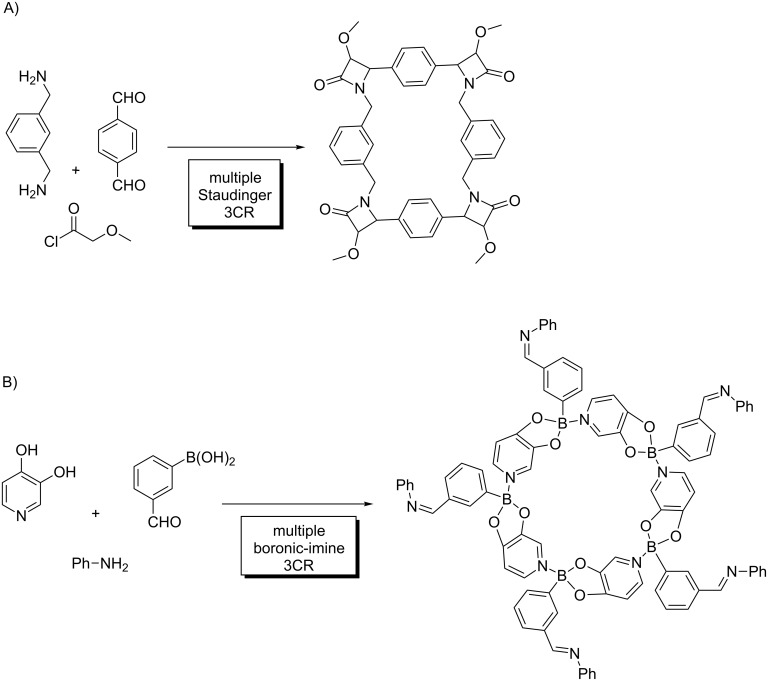
Macrocyclization by MMCRs. A) Staudinger MCR; B) boronic-imine MCR.

This multiple multicomponent macrocyclization strategy [18,20] (Scheme 4) constitutes a breakthrough in the field, providing a powerful synthetic tool to systematically design and prepare a variety of structures. The implementation of this approach goes beyond simple macrocycles, and was exploited to deliver macromulticycles [21], supramolecular structures (cryptands, cages, cryptophanes, podands, etc.) [22–24], cyclic/macrocyclic peptides [25] and other complex structures in a straightforward manner (Scheme 5). The diversity in these systems arises not only from combining a variety of building blocks (from simple aromatic and aliphatic substrates to peptides, steroids, sugars, etc.) [26], but also from the different MCRs used. Although the Ugi 4CR is the most commonly used transformation in this context, interesting examples exploiting other MCRs have been reported (Scheme 6) [27–28].

**Scheme 7:**
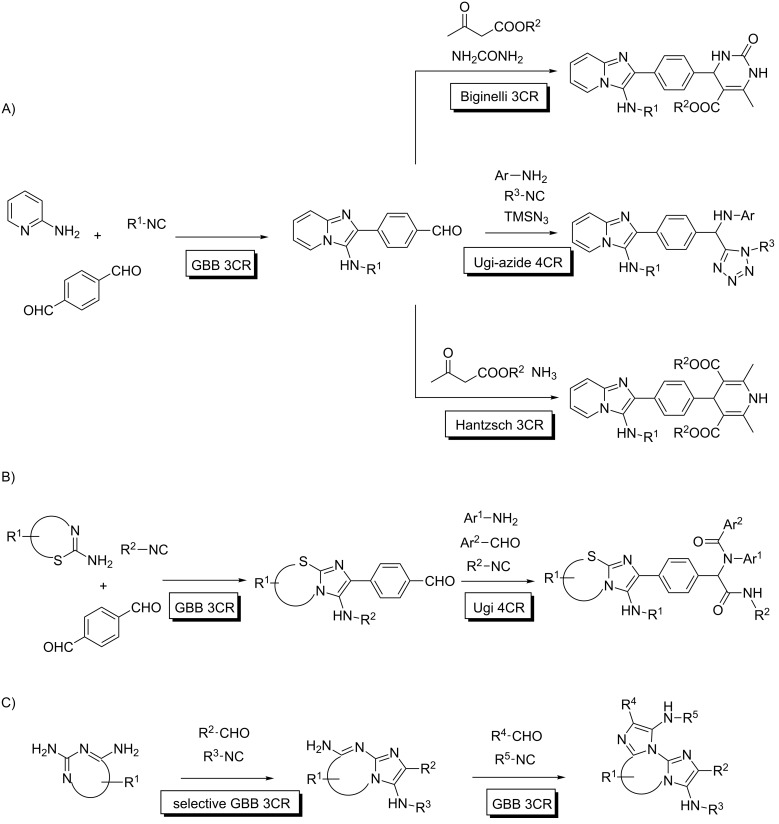
Selective Sequential MMCRs. A and B) MCRs involving terephthalaldehyde; C) Multiple GBB processes with diaminodiazines.

However, when the first MCR step in the process leads to a less reactive intermediate, the system may stop temporarily at this level, and once completed, a second MCR with a distinct set of reagents may take place under more vigorous activation. This allows for selective sequential MMCRs, although there is a clear dependency on the structural features of a given substrate. For instance, all known examples deal with ditopic compounds, where the two reactive FGs are conjugated. In this way, after the initial MCR, the intermediate adduct is somewhat deactivated with respect to the initial substrate, and the remaining FG is less prone to suffer the same transformation. Relevant examples are shown in Scheme 7.

Terephthalaldehyde is capable of undergoing sequential MCRs in a selective manner with two different sets of reactants. In this way, a variety of transformations involving Groebke–Blackburn–Bienaymé (GBB)/Hantzsch, and Biginelli/Ugi-azide sequential reactions were reported by Shahrisa [29]. Similarly, Sharma and co-workers disclosed a related approach [30]. Moreover, 2,4-diaminopyrimidine underwent selective GBB processes leading to a single monoadduct, which reacted again with another isocyanide/aldehyde pair to yield a 5-component adduct in a selective manner. Interestingly, the protocol allows control of the respective localization of all reactant inputs. Thus, inverting the order of the MCRs leads to the complementary disposition of the residues in the final MCR adduct [31].

Non-symmetrically polyfunctionalized components can significantly diversify the synthetic output of MMCRs. However, a limiting factor in the design of MMCRs with such components is the risk of generating undesired cross-adducts. This problem can be avoided by introduction of protecting groups or through the sequential generation of repeating FGs (see below). An alternative strategy is to include those duplicated FGs displaying distinct reactivity. In this way, one FG selectively reacts through the first MCR, while its counterpart remains intact to be exploited in a subsequent transformation.

**Scheme 8:**
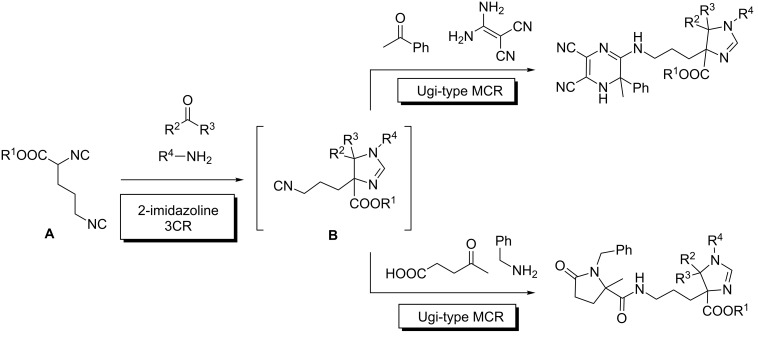
Biased substrates for selective MMCRs.

This elegant concept has been reported by Orru [32], exploiting a non-symmetrical diisocyanide **A** (Scheme 8). The designed sequence requires the α-acidic isocyanide to undergo the 3CR leading to a 2-imidazoline in the first step, the aliphatic isocyanide remaining intact (without protection) and being later incorporated in a variety of isocyanide-based MCRs. In this way, the selective formation of intermediate **B**, leads to the following MCR processes based in different isocyanide MCRs. This approach made possible a remarkable 8-CR process for the one-pot synthesis of compounds with up to 11 diversity points.

**Scheme 9:**
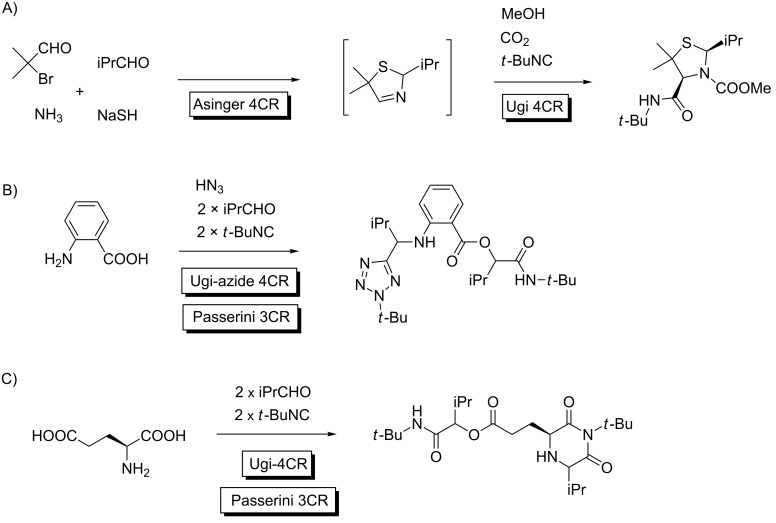
The Union concept. A) Asinger–Ugi combination; B) Passerini–Ugi/azide from anthranilic acid; C) Passerini–Ugi multiple MCR from glutamic acid.

A conceptually distinct approach for selective MMCRs is based in reactivity features. The Union concept (the combination of MCRs) [33–34] is extremely fruitful and was developed by Ugi and Dömling to perform a 7CR out of the combination of Asinger and Ugi transformations (Scheme 9A) [35]. In this context, chemoselectivity can be achieved when the key polyfunctionalized reactants bear the adequate FGs and the combination of MCRs becomes feasible. This can happen either by the orthogonal reactivity of the participating FGs or because the sequential character of the process implies the participation of the first MCR adduct as a reactant in the following transformation. Several examples use this approach with the same set of reagents leading to MMCRs, with limited structural variability (Scheme 9B and C) [36–37]. However, in some occasions the two processes are split up and higher levels of diversity can be achieved.

**Scheme 10:**
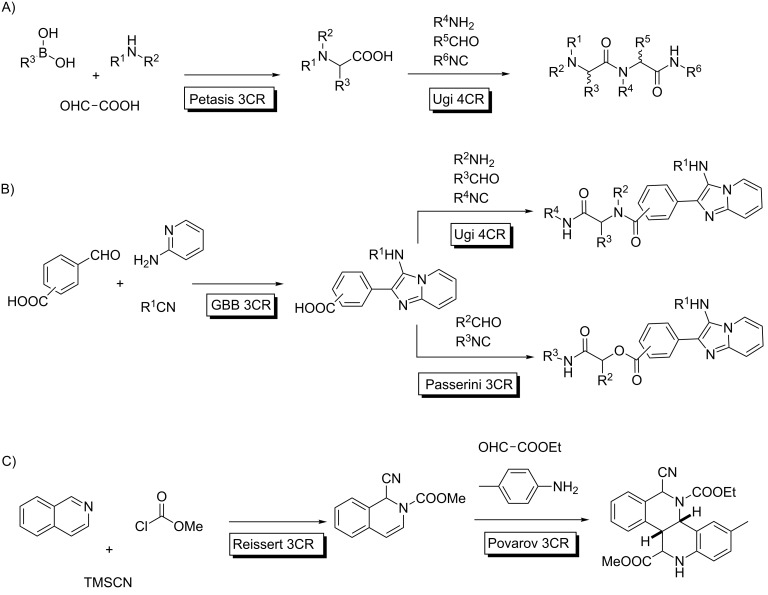
Relevant examples of consecutive MCRs exploiting the Union Concept. A) Petasis-Ugi combination; B) GBB-Ugi/Passerini combination; C) Reissert MCRs linked to a Povarov reaction.

In these cases, the consecutive reactions lead to adducts displaying a diversity of substituents at several positions, which, in principle, can be located at will, in a controlled manner. For instance, the combination of a Petasis 3CR with an Ugi 4CR led to a peptide structure with six diversity points arising directly from the reactants (Scheme 10A) [38]. Similarly, a suitably substituted aldehyde participates in a GBB MCR to yield an adduct which participated in standard Ugi–Passerini processes through the carboxylic acid functional group, untouched in the first MCR (Scheme 10B) [39]. Furthermore, Reissert or Reissert/Ugi reactions can be linked with Povarov MCRs through the intermediacy of the enamine-containing adducts from the former processes (Scheme 10C) [40].

Many additional combinations arise from the Union concept, such as Bredereck–Passerini [41], pyridone-cyclocondensation/Passerini–Ugi [42], azadiene-keteneimine Diels–Alder [43], Asinger–Ugi [44], etc.

**Scheme 11:**
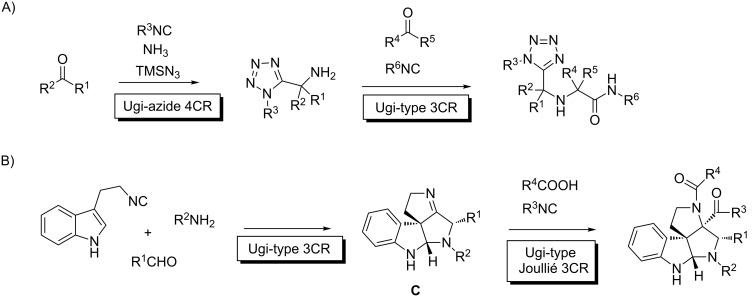
Selective MMCRs featuring FGs with distinct reactivity along the sequence. A) Synthesis of aminomethyltetrazoles by consecutive isocyanide MCRs. B) Interrupted Ugi/Joullié sequence.

Another class of reactions highlight the importance of the distinct kinetic reactivity of similar FGs present in the reactants and in the initial adducts of the first transformation, then enabling productive and selective combinations of the MCRs. The synthesis of aminomethyltetrazoles arising from two consecutive isocyanide-MCRs shows excellent selectivity and broad scope, and although it combines two different transformations, the amine component (ammonia in the starting mixture and a primary amino group in the first adduct) reacts at different rates in each substrate, allowing the controlled performance of both processes, leading to a formal two-step 6CR [45] (Scheme 11A). Analogously, Ruijter reported an interrupted Ugi process [46] involving a 2-(3-indolyl)ethyl isocyanide, an aldehyde and an amine which elegantly led to the iminospiro adduct **C** (Scheme 11B). This compound does not react under the initial reaction conditions, but can be forced to do so in a Joullié MCR with a new isocyanide/carboxylic acid pair [47]. Incidentally, this last process is also remarkably stereoselective, something very unusual in Ugi-type reactions.

**Scheme 12:**
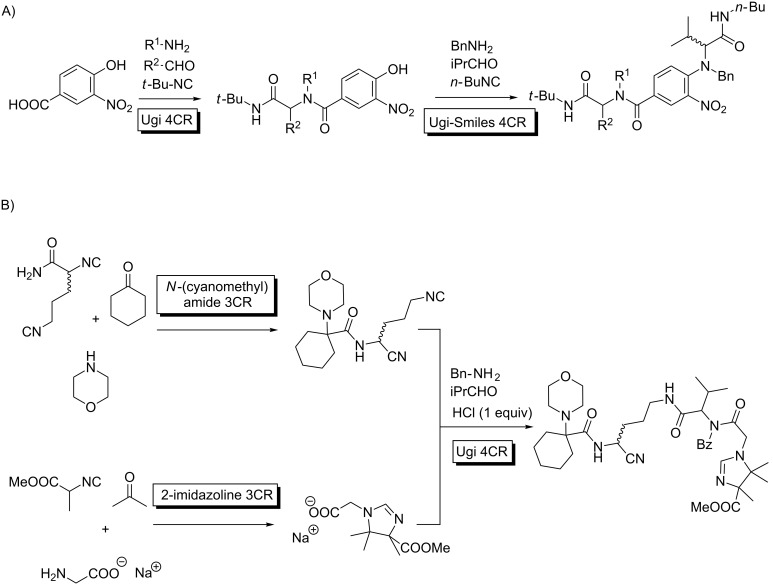
High order MMCRs. A) Ugi/Ugi–Smiles 7C combination; B) imidazoline-*N*-cyanomethylamide-Ugi union leading to an 8CR.

Finally, to exemplify the complexity levels that can be achieved with this approach, we list two multiple MCRs showing their power to reach very elaborate scaffolds with several diversity points in short sequences. Westermann described a remarkable Ugi/Ugi–Smiles protocol using a carboxylic acid provided with a phenol group, which led to a 7-component transformation in a sequential manner (Scheme 12A) [48]. The process can be kinetically justified taking into account that the Ugi MCR with the acid input is much faster than the Ugi–Smiles transformation involving the phenol. In another impressive transformation, a formal 8-component adduct can be assembled through a one-pot protocol combining imidazoline, cyanomethylamide and Ugi MCRs. Although the final product is a complex stereoisomeric mixture, the process stands as a milestone for the rapid construction of complex structures, amenable to combinatorial diversification (Scheme 12B) [32]. Incidentally, the reactivity of the diisocyanide leading to the cyanomethylamide MCR, features a distinct reactivity between the two FGs (see above).

Another scenario involves substrates with different FGs where the selective combination of consecutive MCRs can be carried out by temporarily blocking one of the reactive sites along the process. This sequential approach involves protection/deprotection steps in which one of the building blocks contains a protected FG to be subsequently (and selectively) activated for the following MCRs.

**Scheme 13:**
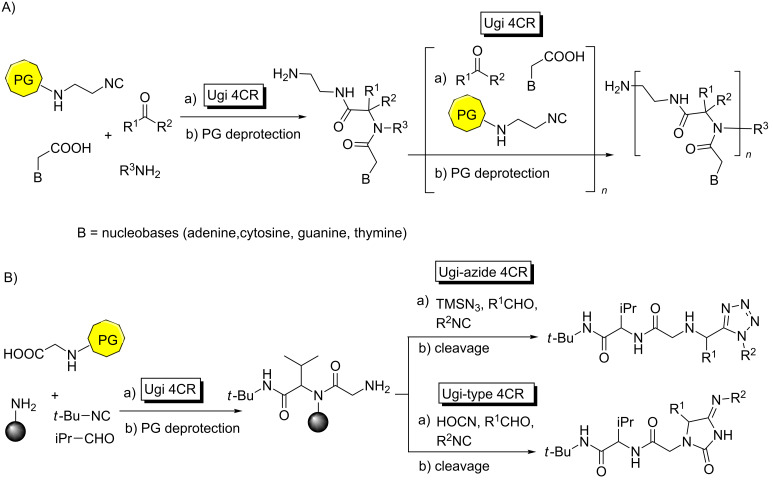
Consecutive Ugi 4CR-deprotection–Ugi 4CR strategy towards A) PNA oligomers and B) peptidic tetrazoles and hydantionimides.

For instance, the examples shown in Scheme 13 illustrate a repetitive Ugi 4CR-deprotection-Ugi 4CR protocol to obtain peptide nucleic acid (PNA) oligomers (Scheme 13A) [49], peptidic tetrazoles and hydantoinimides (Scheme 13B) [50], respectively. Incidentally, the later processes take place in solid phase, which enhances their synthetic suitability.

**Scheme 14:**
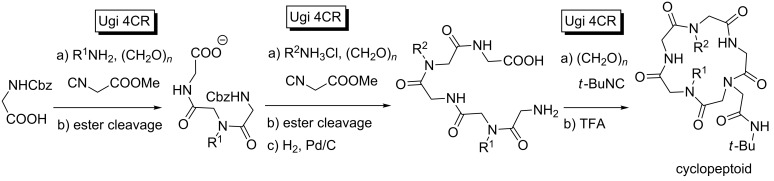
Sequential Ugi 4CR-deprotection access to cyclopeptoids.

The same strategy was exploited by Wessjohann for the synthesis of RGD (Arg-Gly-Asp)cyclopeptoids [51]. In this case, they developed a stepwise protocol in which two Ugi 4CRs, flanking a deprotection, provided linear peptidic adducts. Afterwards, these intermediates were again deprotected and a final Ugi 4CR-macrocyclization efficiently afforded the final target (Scheme 14).

**Scheme 15:**
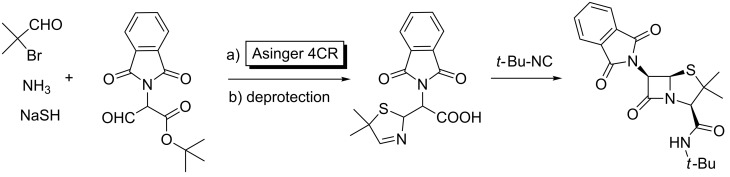
Stepwise access to 6-aminopenicillanic acid derivative through an Asinger, deprotection, Joullié approach.

Furthermore, additional sequential versions of multiple MCRs have been employed to construct natural products. For instance, Ugi reported the synthesis of a 6-aminopenicillanic acid derivative using two different MCRs [33,52]. As shown in Scheme 15, the initial Asinger 4CR yielded an adduct which was selectively deprotected for the following intramolecular Joullié reaction, leading to the penam derivative.

**Scheme 16:**
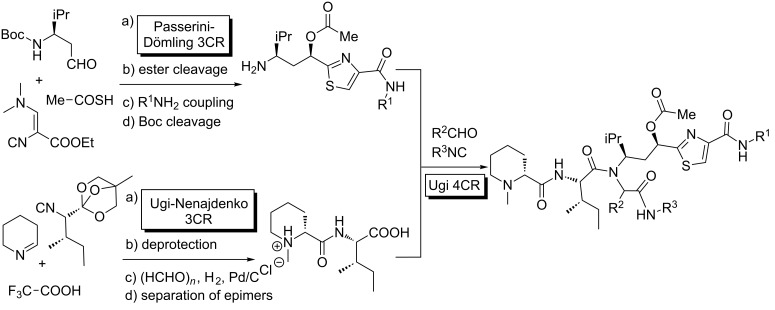
A triple MCR-deprotection approach affording anticancer peptidomimetics.

Finally, an elegant MCR–deprotection strategy was reported by Wessjohann for the synthesis of Tubugis, highly potent antitumor peptidomimetics (Scheme 16) [53]. The convergent approach employs three different isocyanide-MCRs, efficiently prepares the building blocks and combines them, intercalating protecting group cleavages.

## Conclusion

The MMCRs approach represents the most efficient way to build chemical complexity and structural diversity around meaningful scaffolds. When different sets of reactants are involved in these processes, the control of the selectivity becomes a fundamental issue. In this respect, a variety of scenarios can be considered. Innate selectivity (the spontaneous selection of reactants) is unknown and, arguably, it would be difficult to reach under standard conditions. However, this lack of selectivity allows the generation of complex mixtures of adducts, useful in combinatorial chemistry for biological purposes. On the other hand, MCR polymerization takes advantage of this behaviour, and a variety of transformations lead to well-defined macromolecules. An interesting case is found in systems where the reaction conditions and the preorganization of some inputs leads to selective macrocyclization, affording extremely complex MCR adducts in just one step. Rationalization of these processes allows programmed access to a variety of structural types. On the contrary, when di(poly)functionalized substrates display conjugation between the reactive FGs, selectivity may arise. When the initial MCR adduct is less reactive than the starting material, a sequential procedure may lead to the following transformation with different reactants to afford the final adducts in a selective manner. Alternatively, substrates with two chemically distinct FGs of the same kind (i.e., isocyanides) may react at different rates, prompting selectivity, usually in a sequential manner. Moreover, the generation of novel FGs in the course of a given MCR can trigger a new one, then allowing for selectivity in another sequential approach.

Finally, the use of protecting groups in reactants undergoing MCRs leads to multistep transformations which, after suitable deprotections, selectively afford the final adducts. Active research is pursued in the field, aiming at the generalization of the aforementioned concepts and their extension to diverse synthetic outcomes.
